# Blood transfusions: an independent risk factor for the development of Candida infections in critically ill surgical patients

**DOI:** 10.1186/cc9657

**Published:** 2011-03-11

**Authors:** G Burghi, G Ortiz, H Bagnulo

**Affiliations:** 1 Hospital Maciel, Montevideo, Uruguay

## Introduction

Blood transfusions are associated with infectious complications. Despite this, only a few studies link the use of blood transfusions with the development of fungal infections. This study was performed to assess risk factors associated with Candida colonization and infection.

## Methods

A retrospective study including all patients admitted to the ICU due to severe abdominal sepsis or severe pancreatitis between July 2005 and July 2010. Factors analyzed were: shock, insulin use, number of surgeries, mechanical ventilation, days of central catheters, treatment with corticosteroids, parenteral nutrition, red blood cell transfusions, and use of antibiotics. Risk factors for Candida colonization and infection were identified by multivariate logistic regression.

## Results

We analyzed 86 patients with severe abdominal sepsis and severe pancreatitis. Mean age 62 ± 16, SAPS II 47 ± 25, 70% required invasive ventilation, and 61% presented shock. Twenty patients (23%) were colonized by Candida. Independent risk factors for Candida colonization were the use of parenteral nutrition (OR, 3.6; 95% CI, 1.0 to 12.6; *P *= 0.03) and transfusion of at least 4 volumes of red blood cells (OR, 12.8; 95% CI, 2.0 to 79; *P *= 0.006). Seven patients (8%) had invasive candidiasis. Independent risk factors associated with this infection were: prior colonization by at least two sites (OR, 10.6; 95% CI, 1.8 to 61; *P *= 0.008), and transfusion of at least 4 volumes of red blood cells (OR, 9.7; 95% CI, 1.6 to 59; *P *= 0.01). Mortality in the Candida infection group was 71% versus 53% in non-infected nor colonized patients (*P *= 0.3).

## Conclusions

Candida infection is always preceded by colonization. The need for antifungal treatment should be based on the degree of colonization. Restrictive transfusional strategies should be established in these patients to reduce invasive Candida infections.

